# A Dual-Target Microbial Therapeutic Strategy for Treating Metabolic Diseases: Complementary Mechanisms and Clinical Prospects of *Lactiplantibacillus plantarum* and *Akkermansia muciniphila*

**DOI:** 10.3390/metabo16040259

**Published:** 2026-04-13

**Authors:** Si Liu, Mao Wang, Xiaobo Sun, Zhihao Jia, Kuilong Huang

**Affiliations:** 1 College of Pharmacy and Bioengineering, Chongqing University of Technology, Chongqing 400054, China; liusi@cqut.edu.cn; 2 Academy of Chinese Medical Sciences, Henan University of Chinese Medicine, Zhengzhou 450046, China; 3 Cambridge-Suda Genomic Resource Center, Suzhou Medical College, Soochow University, Suzhou 215123, China

**Keywords:** *Lactiplantibacillus plantarum*, *Akkermansia muciniphila*, metabolic diseases, gut microbiome, complementary mechanisms

## Abstract

Metabolic diseases, including obesity, type 2 diabetes, and their related complications, have emerged as major global public health challenges. Increasing evidence indicates that gut microbiota dysbiosis contributes to disrupted metabolic homeostasis, chronic low-grade inflammation, and progression of metabolic disorders. Among candidate microbiome-based interventions, *Lactiplantibacillus plantarum* (*L. plantarum*) and *Akkermansia muciniphila* (*A. muciniphila*) have attracted particular attention because they regulate host metabolism through partially distinct yet potentially complementary mechanisms. *L. plantarum* has been associated with modulation of appetite-related hormones, adipose tissue remodeling, reinforcement of intestinal barrier function, and attenuation of inflammatory signaling. *A. muciniphila* has been linked to strengthening of the mucus barrier, production of beneficial metabolites, and improvement in immune and metabolic homeostasis. However, current evidence remains fragmented across strain-specific studies, heterogeneous formulations, and predominantly single-strain experimental designs, and direct comparative evidence for combined administration is still limited. This review synthesizes current epidemiological, mechanistic, preclinical, and clinical evidence on *L. plantarum* and *A. muciniphila*, with emphasis on their physiological traits, gut ecological adaptability, pathway-based metabolic effects, and translational challenges in obesity, type 2 diabetes, and related complications. We further highlight the ecological rationale for their functional complementarity and discuss priorities for future combination studies and precision implementation. Overall, the available literature supports functional complementarity and possible additive metabolic benefits, but synergistic effects in humans remain unconfirmed. A clearer understanding of strain identity, active therapeutic entities, delivery strategies, and host context will be essential for advancing this dual-target microbial strategy toward clinically meaningful applications.

## 1. Introduction

Metabolic diseases are a group of chronic conditions that are characterized by imbalanced energy intake and expenditure, leading to the dysregulation of energy-supplying substances such as glucose, fatty acids, and cholesterol. According to recent global epidemiological estimates, more than 1.9 billion adults worldwide are overweight, of whom approximately 650 million are living with obesity, while an estimated 537 million adults were living with diabetes globally in 2021 [1,2,3]. Disorders of glucose and lipid metabolism, along with their severe complications—such as atherosclerotic cardiovascular disease, stroke, and metabolic dysfunction-associated steatotic liver disease (MASLD)—represent one of the most significant public health challenges of the 21st century, posing great threats to human health [4]. These disease spectrums arise from a series of interconnected and complex pathological conditions. Metabolic diseases share some similar risk factors, such as chronic inflammation, oxidative stress, and gut microbiota dysbiosis. Although conventional pharmacological treatments and lifestyle interventions can largely alleviate these diseases, they still present certain limitations, including poor patient adherence and notable side effects. These constraints hinder effective disease prevention and treatment, underscoring the urgent need for the exploration and development of alternative approaches.

The human gastrointestinal tract hosts a complex microbial ecosystem, predominantly composed of bacteria along with viruses, fungi, and archaea, which collectively exert profound influences on host physiology, metabolism, and immune function [5]. Recently, the impact of gut bacteria on host homeostasis has garnered increasing attention, both within the gastrointestinal tract and in distant organs. Research has revealed that gut microbes play critical roles in maintaining intestinal barrier integrity and regulating immune responses, significantly influencing health and disease susceptibility [6]. At the genus level, commonly reported gut taxa include *Bacteroides, Bifidobacterium, Ruminococcus, Prevotella, Roseburia*, and *Lactobacillus* [7,8]. At the species level, both *Akkermansia muciniphila* (*A. muciniphila*) and *Lactiplantibacillus plantarum* (*L. plantarum*) are frequently discussed in relation to host metabolism and immunity regulation [9,10,11,12]. An imbalanced gut microbiota has been shown to be linked with a range of conditions, from metabolic disorders like obesity and diabetes to autoimmune diseases and cancer. Multiple studies have characterized microbial signatures associated with increased metabolic disease risk and explored how gut bacteria influence inflammation, metabolic homeostasis, and host susceptibility to disease [13]. This underscores the profound impact of gut microbiota on host health. The intricate relationship between gut microbes and disease development highlights the need for targeted interventions in disease prevention and treatment strategies [14,15].

Among gut microorganisms, strains such as *L. plantarum* and *A. muciniphila* have attracted considerable attention due to their potential roles in metabolic regulation [11,16]. Clinical studies suggest that administering *L. plantarum* TCCC11824 to obese patients can improve body weight and serum low-density lipoprotein cholesterol (LDL-C) outcomes [17]. In contrast, early-stage clinical research indicates that pasteurized *A. muciniphila* can improve selected metabolic parameters in overweight or obese subjects [18]. Notably, beyond interventional studies, population-based and mechanistic evidence further links these organisms to host metabolic phenotypes; in particular, higher *A. muciniphila* abundance is often associated with more favorable metabolic profiles and inversely related to metabolic disease risk [19,20]. Phylogenetically, these organisms belong to Firmicutes and Verrucomicrobiota, respectively, and represent functionally relevant components of the gut ecosystem. Intervention strategies involving these two organisms are often framed as exogenous supplementation in the case of *L. plantarum* and endogenous enrichment or targeted modulation in the case of *A. muciniphila*. Emerging evidence also suggests a potential indirect ecological interaction between them. Multi-omics and mechanistic studies indicate that *L. plantarum*-mediated metabolic remodeling may promote enrichment of *A. muciniphila* through cross-feeding-related processes, thereby providing preliminary support for their potential functional complementarity within the gut ecosystem [21,22].

Despite growing interest in *L. plantarum* and *A. muciniphila* as microbiome-based interventions, several knowledge gaps still limit mechanistic clarity and clinical translation. The evidence base is dominated by single-strain studies and preclinical models, with substantial heterogeneity in strains, dosing regimens, formulations, and endpoints. Moreover, strain-resolved pathways linking microbial outputs such as short-chain fatty acids (SCFAs) and bile acid remodeling to host receptors and downstream signaling pathways—including G protein-coupled receptors 41 and 43 (GPR41/43), the farnesoid X receptor (FXR), Takeda G protein-coupled receptor 5 (TGR5), glucagon-like peptide-1 (GLP-1), and AMP-activated protein kinase (AMPK)—remain incompletely defined. Direct comparative human evidence for combined administration remains limited, and interaction effects beyond additivity are rarely tested. Reported benefits may also arise from distinct bioactive entities, including viable or pasteurized cells, defined surface proteins (e.g., Amuc_1100), extracellular vesicles, or metabolites/secreted factors, each with different stability and dosing implications [18,19,23]. Finally, host context—including habitual diet, baseline microbiota, medications, and comorbidities—likely shapes responsiveness and safety profiles. Accordingly, this review synthesizes epidemiological, mechanistic, and clinical evidence to summarize the physiological traits and gut adaptability of *L. plantarum* and *A. muciniphila*, organize reported benefits within a pathway-based framework for obesity, type 2 diabetes, and related complications, and highlight translational challenges and priorities for future combination trials and precision implementation.

## 2. Review Design and Literature Search

This article is a narrative review that synthesizes preclinical and clinical evidence on *L. plantarum* and *A. muciniphila*, with an emphasis on mechanistic pathways (e.g., SCFAs, bile acid signaling, barrier integrity/endotoxemia, immune–metabolic crosstalk, and enteroendocrine regulation) relevant to metabolic diseases.

To ensure coverage of key lines of evidence, we searched PubMed, Web of Science, Embase and Cochrane library from 2005 to 31 January 2026. Searches combined controlled vocabulary (when available) and free-text key words. A core query combined organism terms with metabolic outcomes: (“*Lactiplantibacillus plantarum*” OR “*Lactobacillus plantarum*” OR “*L. plantarum*”) AND (“*Akkermansia muciniphila*” OR “*A. muciniphila*”) AND (obesity OR “type 2 diabetes” OR “insulin resistance” OR “metabolic syndrome” OR NAFLD OR NASH OR dyslipidemia). Reference lists of eligible articles and relevant reviews were screened to identify additional studies. A total of 117 relevant publications were identified, and the references cited in this review represent key studies supporting the mechanistic synthesis presented herein.

## 3. Physiological Features and Gut Ecological Adaptation of *L. plantarum* and *A. muciniphila*

As a Gram-positive bacterium belonging to the Firmicutes phylum, *L. plantarum* is a versatile microorganism. It possesses unique physiological characteristics and is widely found in natural environments, the human gastrointestinal tract, and various fermented foods [24], allowing it to occupy a distinct ecological niche within the gut microbiome [25]. *L. plantarum* exerts positive effects on host health [26,27], attributable to its antibacterial [27], antioxidant [28,29], and acid tolerance properties [28,30], as well as its capacity to adhere to intestinal surfaces and mucus [27,31]. Its prominent application value in both the food industry and pharmaceutical fields has made it a research focus in recent years. Comparative genomics indicates substantial genome diversity across *L. plantarum* strains and a broad repertoire of carbohydrate-active enzymes and transport systems, supporting flexible utilization of diverse dietary carbohydrates and, in some contexts, host-derived glycans [32,33,34,35,36]. Additionally, it can produce lactic acid along with other metabolites such as alcohols, ketones, and B vitamins. This metabolic diversity confers niche adaptability across different environments and facilitates the establishment of interaction networks with other gut microbiota through mechanisms like cross-feeding [37]. Hussain et al. reported that oral administration of *L. plantarum* LB818 in obese mice restored the gut microbiota composition and promoted the abundance of beneficial bacteria, including *A. muciniphila*, *Bacteroides*, *Bifidobacterium*, and *Lactobacillus* [26]. Certain strains of *L. plantarum* can also secrete broad-spectrum bacteriocins (e.g., plantaricin), which modulate microbial community structure [38]. In the medical field, preclinical studies suggest that selected *L. plantarum* strains may modulate the gut–brain and gut–liver axes in models of neurodegeneration, mood disorders, and liver injury; however, robust clinical evidence for these indications remains limited compared with metabolic endpoints [1,39,40,41].

In contrast, *A. muciniphila*, a representative genus of the Verrucomicrobiota phylum, occupies a distinct ecological niche compared to *L. plantarum* [42]. First isolated from human feces in 2004, it is a specialized mucin degrader that colonizes the intestinal mucus layer. It utilizes mucins secreted by goblet cells as its primary carbon and nitrogen sources, possessing a complete metabolic system dedicated to mucin cleavage [43]. Genomic sequencing reveals an abundance of genes encoding glycoside hydrolases, sulfatases, and sialidases, enabling efficient degradation of complex O-glycosylated mucin structures [44]. Unlike typical commensals that acquire nutrients by co-digesting dietary compounds with the host, *A. muciniphila* adopts a “mucus-adapted, mucin-specialized commensal” lifestyle. Its metabolic activity promotes mucin secretion by goblet cells, maintaining mucin homeostasis and reinforcing the intestinal barrier function [9,18,45]. This barrier enhancement helps prevent pathogen invasion and reduces inflammation. Furthermore, its metabolism generates short-chain fatty acids like acetate and propionate, which nourish colonocytes and participate in systemic metabolic regulation. These mechanisms underpin its observed health benefits. Since the landmark discovery in 2013 that its supplementation alleviates obesity [46], higher *A. muciniphila* abundance has been consistently linked to metabolic health in humans, including improved glucose homeostasis, reduced adiposity, enhanced insulin sensitivity [46,47], and modulated fatty acid and bile acid metabolism [48,49].

Microbial effects in the gut are shaped not only by host signaling but also by microbe–microbe interactions. Accordingly, several reports suggest that *L. plantarum* and *A. muciniphila* may influence each other’s fitness through niche-dependent microenvironmental and metabolic routes—for example, by altering local redox conditions and through substrate cascades involving dietary and mucus-derived glycans, with the balance between facilitation and competition depending on diet and baseline community structure [22,50,51,52]. These ecological considerations provide a conceptual foundation for the pathway-oriented framework presented in the following section.

## 4. Mechanistic Pathways Linking *L. plantarum* and *A. muciniphila* to Metabolic Benefits

### 4.1. Ecological Rationale for Functional Complementarity

Although direct combination trials are still limited, there is growing ecological and experimental evidence supporting functional interaction between *L. plantarum* and *A. muciniphila* in the gut. First, they preferentially occupy partially distinct niches: *L. plantarum* is typically more active in the small intestine and proximal colon lumen where dietary carbohydrates are abundant, whereas *A. muciniphila* is enriched at the colonic mucus layer and specializes in mucin utilization. This spatial partitioning reduces direct competition and enables division of labor across intestinal compartments. Second, *A. muciniphila*-driven mucin turnover can liberate oligosaccharides and monosaccharides that may become available to other commensals (including lactobacilli), while *L. plantarum* can rapidly ferment available substrates to lactate and acetate, which can be further converted by the broader community into butyrate/propionate—metabolites that reinforce barrier function and modulate enteroendocrine signaling. Consistent with this cross-feeding concept, recent multi-omics and mechanistic studies have shown that *L. plantarum* SFF123-mediated metabolic remodeling promotes the enrichment of *A. muciniphila* through tryptophan metabolism and retinoic acid biosynthesis [22]. Additionally, *L. plantarum* LB818 administration in obese mice was reported to increase the abundance of *A. muciniphila*, *Bacteroides*, and *Bifidobacterium*, suggesting a favorable ecological shift that may support mucus-associated communities [26]. Third, *L. plantarum* is aerotolerant and may influence local redox conditions and inflammatory tone, which could indirectly favor mucus-associated anaerobes and stabilize mucus-associated communities. Collectively, these features support a mechanistic model in which the two organisms may be complementary across space, substrates, and timing, providing a rationale for future combination studies that include appropriate monotherapy and combination arms to formally test interaction beyond additivity.

**Figure 1:**
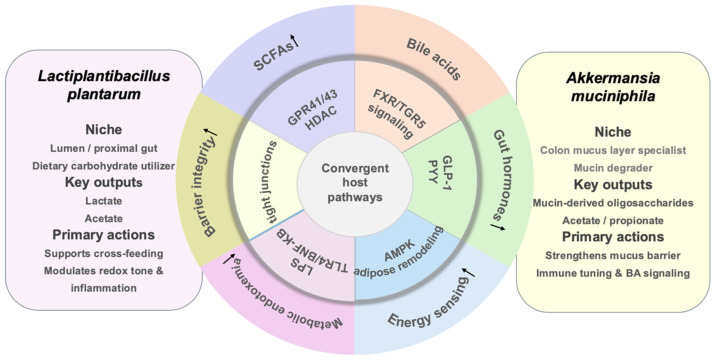
Conceptual framework for complementary (potentially additive) mechanisms of *L. plantarum* and *A. muciniphila*.

Rather than attributing the effects of these two strains to a single mechanism, current evidence points to several recurring host–microbe axes through which they may exert metabolic benefits. These include SCFA production and carbohydrate fermentation [53], bile acid remodeling and FXR/TGR5 signaling [54], intestinal barrier integrity and metabolic endotoxemia [55], immune–metabolic reprogramming [56], and energy-sensing pathways such as AMPK [57]. Figure 1 provides a schematic overview of these convergent pathways.

The schematic highlights niche partitioning, substrate/metabolite cross-feeding, and convergence on key metabolic signaling axes (SCFAs–GPR41/43/HDAC, bile acids–FXR/TGR5, barrier integrity (mucus, tight junctions) and lipopolysaccharide (LPS) translocation, immune–metabolic tuning, gut hormones, and AMPK-related energy sensing). Because direct comparative factorial trials remain limited, this figure presents a hypothesis-generating mechanistic framework grounded primarily in single-strain and preclinical evidence. Arrow direction legend: Upward arrows (↑) indicate an increase or upregulation; downward arrows (↓) indicate a decrease or downregulation.

### 4.2. SCFA-Mediated Signaling

SCFAs, primarily acetate, propionate, and butyrate, serve as key signaling molecules linking gut microbial metabolism to host metabolic health [53]. *L. plantarum* and *A. muciniphila* contribute to SCFA-related effects through distinct substrate preferences and metabolic routes. *L. plantarum* primarily ferments dietary carbohydrates to lactate and acetate, and its genome encodes a broad repertoire of carbohydrate-active enzymes that support flexible carbohydrate utilization [32]. In contrast, *A. muciniphila* degrades mucin-derived glycans and produces metabolites including acetate and propionate [58]. These microbial products can activate receptors such as GPR41 and GPR43 on enteroendocrine cells, thereby promoting GLP-1 and peptide YY (PYY) secretion and influencing appetite regulation and glycemic control [59]. SCFAs also exert histone deacetylase-inhibitory effects, which may dampen inflammation and improve insulin sensitivity. Accordingly, the complementary substrate utilization of these two organisms provides a biologically plausible route for convergent metabolic benefits, although direct evidence from combination studies remains limited. This pathway is summarized schematically in Figure 1.

### 4.3. Bile Acid Remodeling and FXR/TGR5 Signaling

Multiple *L. plantarum* strains display bile tolerance and can influence bile acid composition (including via bile salt hydrolase activity), which may reshape downstream signaling via FXR and TGR5 [54]. Consistent with this axis, strain NCHBL-004 increased active GLP-1 after oral glucose and shifted the gut microbiome toward SCFA- and secondary bile acid-associated functions, consistent with a possible involvement of bile acid–TGR5 signaling, although direct receptor activation was not tested [60]. *A. muciniphila* has also been associated with shifts in bile acid profiles and bile acid–receptor signaling, providing an additional route through which mucus-layer remodeling may couple to enteroendocrine outputs and downstream metabolic phenotypes [61,62]. In turn, bile acid–receptor signaling may serve as a mechanistic bridge between microbiota remodeling, enteroendocrine outputs, and downstream metabolic phenotypes.

### 4.4. Intestinal Barrier Reinforcement and Metabolic Endotoxemia

A recurring feature in metabolic disease models is impaired barrier function with increased translocation of microbial products such as LPS, driving chronic low-grade inflammation. *A. muciniphila* preferentially colonizes the mucus layer and is frequently associated with improved mucus thickness, tighter junctional integrity, and reduced endotoxemia; in parallel, *L. plantarum* has been reported to support barrier repair through effects on tight junction proteins and local inflammatory tone. Framing barrier protection as a measurable intermediate also clarifies how metabolic outcomes can improve without large changes in calorie intake [19,46,55,63,64].

### 4.5. Immune–Metabolic Reprogramming

Both strains have been linked to modulation of innate and adaptive immune signaling, including attenuation of nuclear factor kappa-B (NF-κB)-related inflammatory programs, shifts in macrophage polarization, and reinforcement of regulatory immune responses in relevant experimental settings [56,65,66,67]. In this pathway-oriented framework, the key outcome is not merely “anti-inflammatory”, but attenuation of the metabolically detrimental immune tone that can sustain insulin resistance, adipose tissue dysfunction, and ectopic lipid deposition. Notably, reported immune effects are often context dependent (strain, dose, host background, and tissue), and are best interpreted as mechanistic plausibility rather than universal, clinically confirmed effects.

### 4.6. Enteroendocrine Signaling (GLP-1 and Related Hormones)

Changes in SCFAs and bile acids can converge on enteroendocrine cells to regulate hormone secretion (e.g., GLP-1, PYY, ghrelin), providing a proximal mechanism for altered appetite, postprandial glycemia, and energy handling [68,69,70,71]. In this context, *L. plantarum* may shape the luminal metabolite milieu through carbohydrate fermentation (e.g., lactate production) that supports SCFA-producing networks, whereas *A. muciniphila* can contribute to acetate/propionate pools via mucin-derived glycan metabolism and may also influence bile acid profiles, thereby modulating enteroendocrine outputs. This endocrine route is particularly useful for interpreting why certain strains show benefits in energy intake and glycemic variability even when body-weight changes are modest, because hormone outputs may shift earlier and more sensitively than anthropometric endpoints.

### 4.7. Energy Sensing and Adipose Tissue Remodeling (AMPK and Thermogenic Programs)

Beyond improving gut barrier function and dampening pro-inflammatory immune tone, multiple experimental studies have further linked *L. plantarum* and *A. muciniphila* to enhanced energy expenditure and adipose tissue remodeling; among the proposed mediators, host energy-sensing pathways—exemplified by AMPK—are often viewed as plausible candidates for driving mitochondrial programs and thermogenesis-related transcriptional outputs. AMPK is a canonical regulator of brown adipose activation and mitochondrial programs, providing a mechanistic basis for interpreting reported shifts in lipid handling and white adipose tissue plasticity beyond simple caloric restriction [72]. In support of organism-specific links to this axis, pasteurized *A. muciniphila* has been reported to improve metabolic phenotypes alongside changes consistent with increased whole-body energy handling even when body-weight changes are modest, highlighting that endocrine and metabolic readouts may respond earlier than anthropometric endpoints [73]. Likewise, selected *L. plantarum* interventions in diet-induced metabolic models have been associated with improved lipid metabolism together with modulation of AMPK-related pathways, consistent with a role for cellular energy sensing in adipose and hepatic remodeling [74]. Moreover, *A. muciniphila*-derived acetate has been shown to engage the hepatic AMPK/SIRT1/PGC-1α (sirtuin 1/peroxisome proliferator-activated receptor gamma coactivator 1-alpha) axis in metabolic liver disease models, providing a concrete metabolite-to-signaling route by which microbiota shifts may couple to host energy metabolism [57]. Importantly, however, most mechanistic evidence to date is derived from single-strain and preclinical studies. Well-controlled factorial experiments that include both monotherapies and the combination, and that formally evaluate whether effects exceed additivity remain limited [75].

## 5. Inter-Organ Crosstalk in Metabolic Complications: Beyond Localized Effects

The extension of the regulatory mechanisms described above suggests that these two strains may jointly contribute to multi-organ protection against metabolic complications. Chronic low-grade inflammation serves as a pathological bridge linking metabolic disorders to multi-organ injury. This persistent, mild inflammatory state provides an important pathophysiological basis for complications associated with obesity, diabetes, and related metabolic diseases. Beyond the intestinal environment, *L. plantarum* and *A. muciniphila* have been reported to modulate immune cell function and suppress pro-inflammatory signaling, thereby providing systemic protective signals to distal organs such as the liver, heart, and brain. Notably, however, the relative contribution of each strain and the extent of any additive or synergistic interaction require direct combination studies for confirmation.

The systemic anti-inflammatory effects of *L. plantarum* arise from its ability to modulate key host immune response programs. At the molecular level, strain FRT4 suppresses the overactivation of the classical pro-inflammatory Toll-like receptor 4 (TLR4)/NF-κB signaling pathway through a Foxo1-dependent mechanism, thereby reducing the production of core pro-inflammatory cytokines such as tumor necrosis factor-alpha (TNF-α) and interleukin-6 (IL-6) at their source [76]. Its impact is also evident at the cellular level. Mechanistic studies indicate that selected *L. plantarum* strains or their derivatives can reprogram macrophage states, suppressing M1-associated inflammatory outputs and promoting an M2-like anti-inflammatory phenotype [66,77]. This modulation of innate immune cell states provides a cellular basis for its broad anti-inflammatory effects.

Interestingly, *A. muciniphila* also demonstrates substantial systemic anti-inflammatory potential through modulation of both innate and adaptive immunity. On the innate immune side, it suppresses excessive activation of the NLR family pyrin domain containing 3 (NLRP3) inflammasome, a critical intracellular danger-sensing complex that amplifies inflammatory responses. By inhibiting NLRP3 activation, *A. muciniphila* may attenuate upstream inflammatory amplification [78]. Within the adaptive immune compartment, strain Amuc_1434 has been reported to enhance CD8^+^ T-cell activity, indicating an immunoregulatory effect that may contribute to host defense and immune homeostasis [79]. In addition, *A. muciniphila* has been associated with enhanced regulatory T (Treg) cell responses in some settings, supporting a broader role in immune balance [80]. Collectively, these findings suggest that *A. muciniphila* may help restrain systemic inflammation and support immune homeostasis, which could contribute to protection against inflammation-related metabolic complications.

Based on the mechanisms summarized above, *L. plantarum* and *A. muciniphila* demonstrate distinct protective effects across multiple organ systems. By inhibiting the NF-κB pathway, *L. plantarum* FRT4 mitigates hepatic inflammatory stress, providing a molecular basis for potential benefit in metabolic liver disease, including non-alcoholic fatty liver disease (NAFLD)-related pathology [76]. In the liver, *A. muciniphila* strain Amuc_1100 has been reported to reduce cholesterol levels and ameliorate features associated with progression toward steatohepatitis in preclinical models [81]. Within the cardiovascular system, studies of selected *L. plantarum* strains support cardiovascular benefits through anti-inflammatory and endothelial-protective effects in atherosclerosis-prone mouse models and in patients with coronary artery disease [82,83].

In the central nervous system, *L. plantarum* HEAL9 has been reported to alleviate cognitive and motor impairments in SAMP8 mice, accompanied by reduced astrocyte and microglia activation, lower levels of inflammatory mediators such as interleukin-1 beta (IL-1β) and NLRP3, and decreased amyloid-beta 1-42 (Aβ1-42) accumulation [40]. In a diabetic mouse model, alternate-day fasting was associated with enrichment of *A. muciniphila*, shifts in host–microbe co-metabolites, and improved neuroinflammation and behavioral outcomes [84]. Additionally, two strains of *A. muciniphila*, GMB0476 and GMB2066, were reported to enhance spatial memory performance by activating brain-derived neurotrophic factor (BDNF) signaling [85].Together, these findings support the possibility that *L. plantarum* and *A. muciniphila* may contribute to neuroprotective effects via the gut–brain axis mechanisms. Ultimately, however, these organ-specific anti-inflammatory actions still need to translate into clinically evaluable outcomes. Taken together, these organ-level observations support the plausibility of a shared anti-inflammatory and barrier-centered systems framework, while remaining hypothesis-generating in the absence of robust human combination trials.

In terms of barrier reinforcement, *L. plantarum* and *A. muciniphila* are often discussed as functionally complementary because they preferentially target different barrier layers. Several *L. plantarum* strains (e.g., WH021) primarily support the epithelial (cellular) barrier by upregulating tight-junction components such as occludin and zonula occludens-1 (ZO-1), thereby strengthening intercellular junction integrity in intestinal epithelial cells [2]. In contrast, *A. muciniphila* preferentially resides in the mucus (chemical) barrier by stimulating goblet-cell activity and increasing mucus-layer thickness, helping shield the epithelium from luminal insults [44]. In vivo permeability assays further support barrier benefits for each organism in specific contexts; for example, selected *A. muciniphila* strains and *L. plantarum* interventions have been reported to reduce fluorescein isothiocyanate-dextran (FITC-dextran) translocation and/or restore tight-junction architecture relative to controls, consistent with improved barrier function and reduced translocation of microbial products [86]. Notably, however, direct factorial studies that include each monotherapy arm and the combination and that formally test whether co-administration improves permeability readouts beyond additivity remain limited. Therefore, while a complementary barrier-restorative model is mechanistically plausible, the magnitude and reproducibility of combination benefits require validation across additional models and, critically, in humans.

At the immunomodulatory level, *L. plantarum* and *A. muciniphila* have each been linked to attenuation of pro-inflammatory signaling, potentially supporting complementary immune effects across contexts. For example, selected *L. plantarum* strains can dampen TLR4/NF-κB-related inflammatory programs, consistent with a capacity to reduce excessive innate immune activation [76]. In parallel, *A. muciniphila* has been associated with tolerogenic immune pathways in some settings, including induction of regulatory T cells [80]. In a non-alcoholic steatohepatitis (NASH) model, *A. muciniphila* supplementation improved inflammatory readouts and modulated hepatic immune cell composition, with flow-cytometric evidence consistent with reduced pro-inflammatory macrophage features and a shift toward more reparative macrophage phenotypes [65]. Together, these findings support mechanistic plausibility for immunometabolic benefits, while rigorous factorial studies that directly test whether co-administration yields effects beyond additivity—and clinical confirmation of combination efficacy—remain limited.

Beyond obesity, type 2 diabetes, and NAFLD/NASH, growing preclinical and emerging clinical literature links *L. plantarum* and *A. muciniphila* to phenotypes in immune regulation, cardiometabolic risk, neurocognitive outcomes, and selected oncologic setting, with the strength of evidence varying substantially by strain, disease context, and endpoint [87,88,89,90]. In oncology in particular, *A. muciniphila* has repeatedly been associated with response to immune checkpoint blockade and can modulate the tumor immune microenvironment in experimental models, motivating interest in its use as a biomarker and potential adjuvant in defined contexts [91,92]. Collectively, these observations highlight the pleiotropic nature of host–microbe interactions. Rather than representing universal, strain-specific effects, the therapeutic outcomes are more accurately viewed as disease-contextual manifestations of a limited set of shared mechanistic axes—including barrier integrity, bile acid–FXR/TGR5 signaling, and SCFA-mediated immunometabolism, as summarized in Table 1.

## 6. Evolution of the Intestinal Microbiota During Combined Probiotics and Healthy Lifestyle Interventions

**Figure 2:**
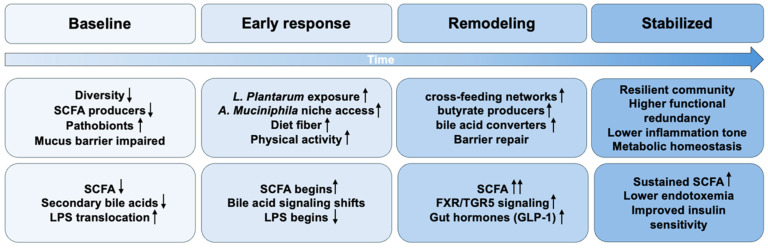
Schematic representation of intestinal microbiota evolution and associated host pathways during combined intervention with probiotics (*L. plantarum* + *A. muciniphila*) and healthy lifestyle intervention. Arrow direction and quantity legend: Upward arrows (↑) indicate an increase or upregulation; downward arrows (↓) indicate a decrease or downregulation. The number of arrows reflects the relative magnitude of change: one arrow (↑ or ↓) indicates a mild change, two arrows (↑↑ or ↓↓) indicates a moderate change.

Figure 2 presents a conceptual, stage-based model describing how the gut microbiota may evolve during a combined intervention that integrates probiotics (*L. plantarum* and *A. muciniphila*) with healthy lifestyle measures (e.g., increased dietary fiber intake and regular physical activity). In the early phase, the model proposes niche engagement and initial metabolic shifts, including increased lactate and acetate availability and mucus-layer-associated colonization. This may be followed by community-level functional remodeling through cross-feeding interactions and enrichment of SCFA-producing taxa. With sustained lifestyle support, microbial functions relevant to host metabolism, such as SCFA production, bile acid signaling through FXR/TGR5, and reduced endotoxin translocation, may become more stable, potentially contributing to improved gut hormone signaling (e.g., GLP-1), lower inflammatory tone, and enhanced insulin sensitivity. This framework is intended to guide hypothesis-driven study design and mechanistic testing; it should not be interpreted as evidence of definitive synergistic effects in humans without direct comparative factorial trials that include each monotherapy arm and the combination.

The diagram illustrates a conceptual progression from baseline dysbiosis, through early response and functional remodeling, to the establishment of a more stable ecosystem, accompanied by sustained improvements in microbial functions, including short-chain fatty acid production, bile acid signaling, reduced lipopolysaccharide/endotoxemia, and downstream host metabolic outcomes.

## 7. Limitations

Several limitations should be considered when interpreting the current evidence base for *L. plantarum* and *A. muciniphila*. First, strain-level heterogeneity is substantial. *L. plantarum* exhibits broad genomic and metabolic diversity, whereas *A. muciniphila* shows strain- and preparation-specific effects (e.g., live vs. pasteurized cells or defined components). Accordingly, results obtained with one strain or formulation should not be assumed to apply to others. This highlights the importance of clear strain identification, genome-informed functional annotation, and dose–response characterization.

Second, the bioactive entity and delivery format remain incompletely standardized. Reported benefits may derive from viable cells, non-viable preparations (e.g., pasteurized *A. muciniphila*), extracellular vesicles, defined surface proteins (e.g., Amuc_1100), cell-wall-associated molecules, bacteriocins, or secreted metabolites, and these may differ in stability, manufacturing requirements, and site of action along the gut. Formulation constraints (e.g., oxygen sensitivity, shelf life, encapsulation, and colon-targeted release) and co-interventions (e.g., dietary fiber, prebiotics, and medications) can strongly influence engraftment and functional readouts.

Third, host context can confound both efficacy and safety. Baseline microbiome configuration, habitual diet, metabolic status, age, and concomitant drug exposure (notably antibiotics and antidiabetic agents) may shape responder phenotypes and the dominant mechanism of action (e.g., SCFA-related effects vs. bile acid signaling). Long-term safety and tolerability—particularly in vulnerable populations—require continued monitoring, and mechanistic claims should be interpreted in light of disease stage and mucosal barrier status.

Finally, direct evidence for combined administration remains limited. Many mechanistic insights are derived from single-strain studies and preclinical models, whereas direct comparative factorial trials, standardized endpoints, and sufficiently long follow-up in humans are scarce. Future studies integrating multi-omics with well-controlled clinical designs will be essential to determine whether the proposed complementarity translates into reproducible clinical benefit.

## 8. Challenges and Opportunities in Clinical Translation

Building on the evidence limitations outlined above, this section focuses on practical translational bottlenecks and emerging solutions. While *L. plantarum* and *A. muciniphila* have shown promising potential in preclinical studies, translation from laboratory findings to clinical application remains challenging. Key barriers include strain-specific functional heterogeneity, formulation stability and delivery efficiency, and limitations in current clinical evaluation frameworks. At the same time, these challenges are driving the development of more precise screening, formulation, and trial-design strategies.

A primary obstacle to effective clinical translation is the functional heterogeneity of bacterial strains. Studies indicate that different *L. plantarum* strains can differ substantially in functional gene content, which may translate into marked differences in metabolic regulatory capacity and antibacterial activity [12,97]. This degree of strain specificity underscores the need for more rigorous strain selection and characterization criteria. Emerging approaches are moving toward more personalized matching strategies, integrating host microbiota profiles, metabolic parameters, and clinical context to improve strain selection and intervention responsiveness, including through machine-learning-assisted prediction frameworks [98,99].

A second closely related barrier is the inconsistent definition of the active therapeutic entity across studies. In practice, “probiotic” and “postbiotic” interventions may involve distinct pharmacological mechanisms: live *L. plantarum* preparations may act through transient engraftment and metabolite production, whereas biological activity may also arise from non-viable biomass, secreted factors, or purified components. For *A. muciniphila*, pasteurized preparations, specific molecules such as Amuc_1100, as well as extracellular vesicles have all been implicated as functional effectors in preclinical studies [18,19,100]. Clear reporting and standardization of what is administered, including strain identity, viability status, dose units, and quality-control specifications are essential for reproducibility, safety evaluation, and regulatory translation.

Despite the growing body of mechanistic evidence, clinical translation remains constrained by formulation and delivery. For strict anaerobes such as *A. muciniphila*, maintaining viability during production and storage, while preserving resistance to gastric and bile stress during delivery, remains a central formulation challenge. Microencapsulation and drying-based stabilization have been shown to improve survival during storage and simulated upper gastrointestinal transit, supporting the feasibility of viable-cell delivery under physiologically relevant stress conditions [101,102]. More advanced microencapsulation strategies, including multilayer systems that integrate oxygen/anaerobic protection, pH-responsive release, and mucoadhesive retention, are increasingly being developed to improve delivery efficiency and intestinal persistence [103,104]. Notably, pasteurized *A. muciniphila* can retain bioactivity in experimental and clinical contexts, supporting the development of stable non-viable formulations or postbiotic products [18,19]. Regulatory assessments have further supported the feasibility of pasteurized preparations as stable products for human use [105]. Collectively, these formulation advances are consistent with the integrated pathway model summarized in Figure 1.

On the regulatory side, requirements for live biotherapeutic and microbiome-based products are evolving, with increasing emphasis on strain identity, potency characterization, genomic stability, and product consistency. These requirements are directly relevant to the translational development of *L. plantarum* and *A. muciniphila* formulations [18,106,107]. Looking ahead, progress will likely depend on clearer postbiotic product definitions, engineered functional strains enabled by synthetic biology, and more individualized delivery strategies [108,109].

Clinical evidence is gradually expanding but remains heterogeneous. Early-phase studies suggest that pasteurized *A. muciniphila* may improve selected metabolic parameters in overweight or obese adults [108]. In addition, emerging trials of *A. muciniphila* supplementation in overweight/obese individuals with T2D suggest metabolic benefits in subgroups, with efficacy depending on baseline abundance [110]. For *L. plantarum*, recent randomized evidence suggests possible modest improvements in LDL-C and total cholesterol in some populations, while pooled analyses indicate that effects on glycemic and lipid outcomes are often small and strain- and population-dependent [111,112,113]; however, some strain-specific RCTs (e.g., LMT1-48) have reported reductions in body-fat-related outcomes in selected cohorts [114].

Safety considerations also require careful attention, particularly for long-term use and in vulnerable populations. Although lactobacilli have a long history of use and are widely regarded as safe in general populations, bacteremia and sepsis attributable to certain *Lactobacillus* strains have been reported in immunocompromised or critically ill patients [115]. In parallel, context-dependent risks have been reported for *A. muciniphila* in specific experimental settings, where host genetic background, microbiota configuration, dietary context, or coexisting disease may influence outcomes [116,117]. These observations support the need for more comprehensive safety evaluation, careful patient stratification, and long-term monitoring during clinical translation.

## 9. Conclusions

This review synthesizes current evidence on *Lactiplantibacillus plantarum* and *Akkermansia muciniphila* as a dual-target microbial strategy for metabolic disease intervention. By organizing findings around key host metabolic pathways, including SCFA-related signaling, bile acid–FXR/TGR5 axes, gut barrier integrity, and immune–metabolic crosstalk, we highlight how these organisms may exert complementary functions across the gut–liver–adipose and gut–brain axes.

However, most mechanistic insights are derived from single-strain studies and preclinical models, and direct comparative evidence for combined administration in humans remains limited. Accordingly, the current literature supports functional complementarity and possible additive effects, rather than confirmed synergistic effects in humans.

Future work should prioritize well-controlled comparative factorial trials with standardized strain identification, explicit active-entity definitions (e.g., live cells, pasteurized preparations, extracellular vesicles, and defined molecular effectors), and harmonized endpoints. Integrating multi-omics with organoid and gnotobiotic models may further clarify context dependence, safety, and whether combination effects can exceed additivity under specific biological or clinical conditions.

## Figures and Tables

**Table 1:** Reported implications of *L. plantarum* and *A. muciniphila* in non-metabolic disease areas (representative evidence).

Disease Area	Reported Direction of Effect	Evidence Level	Key Mediators/Axes	Representative References
Cardiovascular disease/atherosclerosis	Improved lipid/inflammation profiles; reduced plaque burden in models	Preclinical; human association	SCFAs; bile acids; barrier–LPS; vascular inflammation	[83,93]
Type 1 diabetes/autoimmunity	Delayed onset or reduced severity in models	Preclinical	Barrier integrity; immune tolerance (Treg/Th1 balance); endotoxemia	[6,80]
Inflammatory bowel disease (e.g., UC)	Attenuated colitis activity in models/clinical adjunct settings	Preclinical; limited human	Mucus layer support; tight junctions; anti-inflammatory cytokine shifts	[5,94]
Neurocognitive disorders (AD-like impairment; memory)	Improved cognitive readouts in animal models	Preclinical	Gut–brain axis; inflammation; SCFA signaling; neurotrophin pathways	[40,85]
Cancer/immunotherapy sensitization	Enhanced anti-tumor immunity in models	Preclinical; translational rationale	Immune–metabolic tuning; CD8^+^ T cell activity; microbial metabolites	[79]
Pregnancy complications (e.g., preeclampsia)	Improved inflammatory/vascular features in models	Preclinical	Inflammation; endothelial function; barrier–LPS axis	[95]
Toxicant-related injury/bone metabolism	Mitigated injury phenotypes in animal models	Preclinical	Immune modulation; oxidative stress; metabolite signaling	[96]

## Data Availability

No new data were created or analyzed in this study.
